# Mechanisms of abnormal adult hippocampal neurogenesis in Alzheimer’s disease

**DOI:** 10.3389/fnins.2023.1125376

**Published:** 2023-02-15

**Authors:** Yujuan Zhou, Xu Wang, Yingying Liu, Yulu Gu, Renjun Gu, Geng Zhang, Qing Lin

**Affiliations:** ^1^Key Laboratory of Brain Aging and Neurodegenerative Diseases, Fujian Medical University, Fuzhou, China; ^2^Department of Physiology and Pathophysiology, Health Science Center, Peking University, Beijing, China; ^3^School of Chinese Medicine and School of Integrated Chinese and Western Medicine, Nanjing University of Chinese Medicine, Nanjing, China; ^4^Laboratory of Clinical Applied Anatomy, School of Basic Medical Sciences, Fujian Medical University, Fuzhou, China

**Keywords:** adult hippocampal neurogenesis, Alzheimer’s disease, energy metabolism, epigenetics, neuroinflammation

## Abstract

Alzheimer’s disease (AD) is a degenerative disease of the central nervous system, the most common type of dementia in old age, which causes progressive loss of cognitive functions such as thoughts, memory, reasoning, behavioral abilities and social skills, affecting the daily life of patients. The dentate gyrus of the hippocampus is a key area for learning and memory functions, and an important site of adult hippocampal neurogenesis (AHN) in normal mammals. AHN mainly consists of the proliferation, differentiation, survival and maturation of newborn neurons and occurs throughout adulthood, but the level of AHN decreases with age. In AD, the AHN will be affected to different degrees at different times, and its exact molecular mechanisms are increasingly elucidated. In this review, we summarize the changes of AHN in AD and its alteration mechanism, which will help lay the foundation for further research on the pathogenesis and diagnostic and therapeutic approaches of AD.

## 1. Introduction

Alzheimer’s disease (AD), also known as senile dementia, is a degenerative disease of the central nervous system with age-related cognitive and functional decline that can eventually lead to death, with an insidious onset and a chronic progressive course, and is the most common type of dementia in old age (Alzheimer’s Association, 2019). The main pathological manifestations of AD are amyloid β-protein (Aβ) deposition, neurogenic fiber tangles due to Tau protein hyperphosphorylation, and neuronal loss (Lei et al., 2021). The clinical manifestations of AD patients are characterized by comprehensive dementia, such as memory impairment, aphasia, loss of use, loss of recognition, impairment of visuospatial skills, executive dysfunction, and personality and behavioral changes. Mild cognitive impairment is the precursor stage of dementia, characterized by subjective cognitive deficits and objective memory impairment in daily life (Xie et al., 2021). Patients have difficulties in reasoning, abstract concepts, and even language difficulties. And, patients may also experience increased manifestations of depression, sleep difficulties and anxiety. According to the Alzheimer’s Disease International assessment, 75% of people with dementia worldwide are undiagnosed, and it can be as high as 90% in some low- and middle-income countries. Based on the latest data from the World Health Organization, the number of people suffering from dementia is estimated to be 55 million in 2019 and expected to increase to 139 million in 2050 (Gauthier et al., 2022). Therefore, it is crucial to study the pathogenesis of AD to find diagnostic methods and the treatment strategies.

In-depth studies of postmortem AD patient brains or transgenic AD mouse models have shown that the neuropathology of AD brain is mainly characterized by neuronal cell death, aggregation of neurogenic fiber tangles and formation of neural plaques (Kou et al., 2020; Rahman et al., 2020). Of these, the aggregation of plaques and fiber tangles in the hippocampus are closely associated with cognitive decline. Unlike other brain regions, the adult mammalian hippocampus contains Neural Stem Cells (NSCs), which can generate new neurons, a process known as Adult Hippocampal Neurogenesis (AHN) (Babcock et al., 2021).

For decades, it was widely believed that the death and loss of neurons was permanent and irrecoverable. However, thanks to continuous efforts in the field of neuroscience, it is now generally accepted that certain areas of adult human brain do undergo neurogenesis during a long lifetime (Kumar et al., 2019), which can produce new functional neurons. The Subgranular Zone (SGZ) in the dentate gyrus (DG) portion of the hippocampus is one of the sites where neurogenesis exists in adult human brain, a process known as AHN. Most NSCs in the adult brain are quiescent and are not active in proliferating, whereas NSCs in the SGZ and the Subependymal Ventricular Zone (SVZ) have the ability to self-renew, divide and differentiate into mature granule neurons (Imayoshi et al., 2008).

**FIGURE 1:**
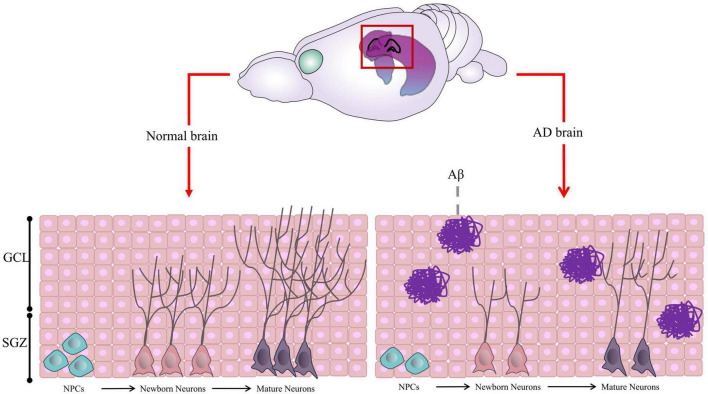
Graphic illustration of Adult Hippocampal Neurogenesis (AHN). In normal brain, the neural precursor cells (NPCs) located in the Subgranular Zone (SGZ) proliferate and differentiate to form neural progenitor cells or neuroblasts, which further differentiate into new neurons. The newborn neurons continue to migrate, proliferate, develop axons and dendrites, become mature neurons, and integrate into the hippocampal network. However, in AD brain, the proliferation and differentiation of neurons are inhibited due to Aβ aggregation and other factors in most cases. Some of this illustration was started from a Scidraw Template.

AHN is a complex process with multiple steps (Figure 1), including (1) Neural Precursor Cells (NPCs) in the SGZ, origin of neurons, divide to be new NSCs or neural progenitor cells; (2) Both NSCs and neural progenitor cells have the ability to proliferate and differentiate into new neurons; (3) Newborn neurons go into the granule cell layer to grow axons and dendrites, eventually connecting with the internal olfactory cortex and hippocampus and integrating into the hippocampal network (Kempermann et al., 2003, 2015; Babcock et al., 2021; Gillotin et al., 2021).

Long-term studies in rodents show that the proportion of NSCs dividing actively in DG region becomes less with age, indicating that neural regeneration declines significantly with age. Like rodents, hippocampal neurogenesis in humans persists with age, but the amount of neurons decreases significantly (Boldrini et al., 2018).

Sustained hippocampal neurogenesis in adults is important for neuronal plasticity, learning and memoring, and emotion regulation, and therefore the importance and relevance of AHN in AD cannot be overstated (Kempermann et al., 2018). In this review, we will summarize the changes of AHN in AD and its alteration mechanisms, laying the groundwork for further investigation into the pathogenesis and diagnostic approaches of AD.

## 2. Altered adult hippocampal neurogenesis in Alzheimer’s disease

Although AHN still exists, its regeneration will decrease with age (Boldrini et al., 2018). As for AD patients, “what is the status of the AHN?” During a long period of research, different voices appeared.

### 2.1. The increase of AHN

More than a decade ago, studies on the effects of AHN in AD mostly based on the brains of deceased AD patients and AD mice model. These findings showed an increase of AHN in the older AD patients, based mainly on the upregulation of the expression of the neuronal markers DCX, neurogenic differentiation factor TUC4 and PSA-NCAM (Jin et al., 2004b). Scientists believe that such an increase of AHN is a compensatory mechanism overcoming neuronal damage due to neurodegeneration (Boekhoorn et al., 2006). To study the occurrence and development of AD, hundreds of AD animal models are available for scientific research. Studies have shown that an increase of AHN in APP_Sw,Ind_ AD model mice (Jin et al., 2004a). In addition, increased neurogenesis has been observed in PS-1 overexpressing and APP/PS1 double transgenic AD mice with disease progression (Zeng et al., 2016).

### 2.2. The decrease of AHN

However, with our deep understanding of the pathogenesis of AD disease and brain networks, different research results have been presented. Comparison between NSCs in brain tissue of AD deaths and that of the control group found that the AD group’s cell survived less, or failed prematurely (Lovell et al., 2006). Even at early stages of Braak staging, when the levels of Aβ and neurogenic fiber tangles accumulation were low, the expression of DCX, SOX2, and MAP2A in the hippocampus were decreased dramatically in AD patients compared with the control group, indicating downregulation of hippocampal neurogenesis (Li et al., 2008; Crews et al., 2010). Mushashi is an evolutionarily conserved RNA-binding protein and stem cell marker that regulates neurogenesis by altering the balance of self-renewal and differentiation (Velasco et al., 2019). When Mushashi and Ki-67 were used as markers in the AD patients, it is found that the number and viability of neural progenitor cells in SVZ were reduced significantly (Ziabreva et al., 2006; Velasco et al., 2019). And a cross-sectional study of several AD patients aged 52–97 found that a relative decline in the number of immature neurons at all stages, compared to healthy older adults. In particular, although DCX-positive cells decreased with age, the decline in AD patients was more severe (Moreno-Jiménez et al., 2019; Tobin et al., 2019). In the study of AD animal models, the SGZ and SVZ regions of 3xTg mice showed significantly impaired neurogenesis, with a 63% decrease in hippocampal neural stem cell proliferation at the age of 4 months and almost no new neurons at the age of 12 months (Rodríguez et al., 2008, 2009; Hamilton et al., 2015). For 5xFAD mice, DCX positive expression in the hippocampal region began to decline at the age of 2 months, and was almost undetectable by the age of 7 months, indicating reduced hippocampal neurogenesis in the mice (Moon et al., 2014).

The differences in findings for AD patients may be due to limitations in sample size or individualized differences that may be accompanied by other diseases. The contradiction between different genotypes of mice may also be the result of the way in which the model mice are induced leading to their different effects on AHN. However, some studies show that for the early stages of AD pathological development, the immune system in the brain still plays a role in improving the microenvironment of the brain by removing cellular debris, which favors AHN. Whereas, as AD disease progresses, the inflammatory response in the brain increases, exacerbating neurotoxicity and decreasing AHN levels (Song et al., 2012; Gray et al., 2020).

In summary, the AHN shows different degrees of changes during the development of AD disease (Table 1). The underlying mechanisms of AHN alteration have also been studied more extensively.

**TABLE 1:** Summary of adult hippocampal neurogenesis in AD patients and model mice.

Sample		Group	Changes in AD hippocampal structure	Neurogenesis marker expression	References
AD patients		Control (*n* = 11),AD (*n* = 14)	The number of new neurons in CA1 region increased	DCX↑,PSA-NCAM↑	Jin et al., 2004b
		Control (*n* = 10),AD (*n* = 9)	The cross-sectional area of CA1 and CA2 zones decreased	Ki67↑,GFAP↑	Boekhoorn et al., 2006
		Control (*n* = 15),AD (*n* = 14)	—	MAP2a/b↑,	Li et al., 2008
		Control (*n* = 5),AD (*n* = 14)	—	DCX↓,SOX2↓	Crews et al., 2010
		Control (*n* = 7),AD (*n* = 7)	Neural progenitor cells in SVZ region were significantly reduced	Musashi1↓,Nestin↑	Ziabreva et al., 2006
AD models	APPTg mice	*n* = 4	Dense amyloid deposits	BrdU↓,DCX↓,SOX2↓	Crews et al., 2010
	Tg2576 mice	—	—	BrdU↓,DCX↓	Krezymon et al., 2013
		—	—	BrdU↓,DCX↓,NeuN↓	Li et al., 2015
	APP_Sw,Ind_ mice	—	—	BrdU↑,DCX↑	Jin et al., 2004a
	PS1 mice	—	—	BrdU↓,NeuN↓	Wang et al., 2004
		—	—	BrdU↓,Calretinin↓	Wen et al., 2004
	3xTg mice	*n* = 3∼7	New neurons appear in the dentate gyrus	HH3↓	Rodríguez et al., 2008
		—	—	DCX↓,Ki67↓,GFAP↓	Hamilton et al., 2015
	5xFAD mice	—	—	DCX↓,HH3↓,Calretinin↓	Moon et al., 2014
		Tg (*n* = 12),non-Tg (*n* = 12)	—	Ki67 (—),DCX↓	Zaletel et al., 2018
		—	—	DCX↑,Ki67↑	Ziegler-Waldkirch et al., 2018
	APP/PS1 mice	—	—	BrdU↓,DCX↓	Demars et al., 2010
		—	—	BrdU↓,Nestin↓	Zeng et al., 2016
		—	—	BrdU↓,DCX↓	Demars et al., 2010

DCX, doublecortin; PSA-NCAM, polysialated form of neural cell adhesion molecule; Ki67, proliferative marker; GFAP, glial fibrillary acidic protein; SOX2, SRY (sex determining region Y)-box 2; BrdU, 5-Bromodeoxyuridine; HH3, Histone H3.

## 3. Key mechanisms of altered AHN in Alzheimer’s disease

Among the major pathogenesis of AD, one of the important hypotheses is the amyloid hypothesis, which states that there is an abnormal accumulation of Aβ in the brain tissue. These peptides are neurotoxic and eventually lead to neuronal death and degeneration, with the hippocampus being the “hardest hit” area (Santin et al., 2011). The hippocampus is the main area where adults continue to produce new neurons. This AHN is closely related to memory, learning, and cognitive abilities in AD patients. With the increasing number of studies on the mechanisms of hippocampal neurogenesis, more is known about how ANH plays its role during the development of AD disease.

### 3.1. Energy metabolism

#### 3.1.1. Glycolysis

Glucose is the only source of energy in the brain. When the utilization of glucose in the brain is impaired, the function of brain will be affected to a large extent (Vercruysse et al., 2018). In a healthy brain, glucose metabolism is an important source of energy for neurons. And when glucose utilization in the brain decreases, so does human cognitive ability (Tingley et al., 2021). In total, 30% of glucose used by the human brain is aerobic glycolysis (Magistretti and Allaman, 2015). Astrocytes are the most abundant cell type and major consumer of glucose in the central nervous system (CNS). Glycolysis and lactate production are metabolic features of astrocytes, while neurons meet their energy requirements mainly through oxidative phosphorylation (Bélanger et al., 2011; Magistretti and Allaman, 2015). Aerobic glycolysis of astrocytes provides important nutritional support for CNS neurons. It has been shown that SNX27^*R*196*W*^ (equivalent to human R198W) attenuates glucose uptake by astrocytes *via* Glucose Transporter 1 (GLUT) in mice, and causes a decrease in lactate production and deficits in synaptic function and learning behavior (Zhang et al., 2022).

NSCs in the SVZ and SGZ regions undergo various stages in the process of differentiation into mature neurons. In this process, stem cells rely on glycolytic metabolism rather than mitochondrial oxidative phosphorylation (Folmes et al., 2011). Mitochondria manage metabolites and the epigenetic state of NSCs, regulating the differentiation process of NSCs during AHN (Khacho et al., 2019). In AD, excessive aggregation of Aβ in the brain induces mitochondrial dysfunction in neural progenitor cells and inhibits the differentiation of newborn neurons by reducing glucose utilization, leading to AHN dysregulation (Kim et al., 2022). In addition, abnormal insulin metabolism is another essential feature of AD, and insulin resistance mainly affects glucose metabolism of the brain in the hippocampus (Watson and Craft, 2003). The reduction of glucose transport and uptake may be caused by GLUT deficiencies. The GLUTs distributed in the brain are mainly GLUT1 and GLUT3, with GLUT1 involved in glucose transport to brain glial cells and GLUT3 transporting glucose to neurons. It has been demonstrated that metformin can normalize glucose transport and uptake in the brain by inhibiting the activity of GSK-3β and increasing the expression of GLUT1 and GLUT3 to promote neuronal regeneration and prevent neuronal damage (Pilipenko et al., 2020).

#### 3.1.2. Lactate metabolism

In addition to important substrates of energy metabolism such as glucose and ketone bodies that provide energy to the brain, lactate metabolism also plays a crucial role in memory. Under high energy demand, lactate is involved in regulating neurogenesis-related processes such as angiogenesis, neuronal excitability and plasticity. Moreover, lactate is also involved in regulating the metabolism and signaling pathways of non-neural cells in the neurogenic microenvironment, such as endothelial cells, oligodendrocytes, microglia and astrocytes (Matsui et al., 2017; Lev-Vachnish et al., 2019). Among the large number of metabolites consumed by oligodendrocytes, in addition to glucose, there is also important lactate, which is used for myelin formation or myelin repair after demyelination injury to promote axonal integrity. Furthermore, the addition of lactate under low glucose conditions can rescue oligodendrocyte lineage cells and promote their myelin formation. It has been suggested that oligodendrocyte dysfunction and early demyelination in APP/PS1 mice may accelerate the progression of AD disease (Ichihara et al., 2017). And lactate treatment produces more myelin compared to glucose, suggesting that oligodendrocytes may be more dependent on lactate as a substrate for myelin production (Rinholm et al., 2011). Some studies have shown that when demyelinating lesions occur in mice, the expression of p-CREB and BDNF will be significantly reduced, further affecting the development of mature neurons in mice, and damaging AHN (Hahn et al., 2022).

Lactate metabolism in the CNS is categorically inseparable from the process of exploring lactate transport, and there are 1–14 isoforms of Monocarboxylate Transporter (MCT), which are distributed in different cells and play different roles. In the central nervous system, MCT1, MCT2, and MCT4 facilitate lactate transport between nerve cells (Pierre et al., 2000). Lactate transport is required for memory formation and synaptic transmission in the hippocampus. When MCT transport is dysfunctional, it may lead to impaired lactate transport, which in turn hinders energy metabolism and causes memory impairment (Nagase et al., 2014). In endothelial cells, the PI3K/AKT pathway regulates MCT-1 expression and subsequent lactate transport, and the transported lactate activates HCAR1 on astrocytes to regulate vascular endothelial growth factor (VEGF) levels, thereby enhancing angiogenesis and promoting AHN. Thus, abnormal lactate accumulation in the hippocampus disrupts AHN and impairs learning and memory functions (Scandella and Knobloch, 2019). According to the astrocyte-neuron lactate shuttle hypothesis, astrocytes promote glucose consumption or glycogen breakdown to enhance lactate production, which is then transported to neurons *via* MCT to support neuronal activity. In AD, the astrocyte’s ability to take up glucose is diminished and lactate production is reduced, which affects the subsequent neuronal regeneration process (Nicola and Okun, 2021). In the course of neuroinflammation, MCT1 dysfunction and changes in lactate levels affect the pro-inflammatory response of microglia. Microglia can phagocytose and remove apoptotic cells, including neoplastic neurons through phagocytosis to further balance the proliferation and survival of neoplastic neurons (Kong L. et al., 2019).

A growing body of research suggests that voluntary physical exercise can upregulate specific neurotransmitters levels in the brain, including the brain-derived neurotrophic factors (BDNF) and VEGF, and increase adult hippocampal neurogenesis, in which lactate plays an important role (Vivar and van Praag, 2017). During physical exercise, lactate concentration in the blood increases, which facilitates neuronal differentiation and survival of newborn neurons (Lev-Vachnish et al., 2019). But how this relationship develops in AD requires further research.

#### 3.1.3. Lipids metabolism

Extracellular aggregation of neuritic plaques and intracellular aggregation of neurofibrillary tangles are histopathological hallmarks of AD and are closely associated with synaptic defects and neurodegeneration (Yamaguchi et al., 1991). Amyloid Precursor Protein (APP) is sheared by β-secretase and γ-secretase to form Aβ fragments, which form neuritic plaques in the brains of AD patients. Both β-secretase and γ-secretase are intact lipoproteins, and their action depends mainly on the level of lipids present in the membrane (Campos-Peña et al., 2022). Evidence suggests that AHN disorders caused by lipid metabolism disorders make patients more susceptible to AD. Monoacylglycerol lipase (Mgll), a hydrolase that breaks down endogenous fats, is now considered a potential target for AD. It was demonstrated that in postmortem AD patients and 3xTgAD mouse models, there is an age-dependent induction of Mgll due to diminished aPKC-CBP signaling, thereby reducing neuronal production in the brains of AD patients and impairing their cognitive function (Syal et al., 2020).

It is well known that cholesterol is the main protein required to maintain the dynamic homeostasis of cell membranes, and the brain is the organ with the highest cholesterol content. It has been demonstrated that cholesterol homeostasis affects the production of amyloid protein. In addition, there are many lipids present in the cell membrane, such as sphingomyelin and cardiolipin, which may alter the function of a variety of transmembrane proteins (Fabiani and Antollini, 2019). In the adult brain, 70–80% of cholesterol is found mainly in the myelin sheath, with the rest in the plasma membrane of astrocytes and neurons, protecting their morphologies and synaptic transmission processes (Dietschy and Turley, 2004; Zhang and Liu, 2015). High levels of cholesterol in the cell membrane also cause significant changes in the dynamic homeostasis of neurons, which affects the proteolytic activity of γ-secretase (a transmembrane protein), including promoting amyloidogenesis, increasing APP processing, producing amyloid, and affecting the microenvironment of AHN (Kao et al., 2020). ApoE is a glycoprotein mainly secreted by astrocytes, which is involved in regulating lipid transport, synaptogenesis and amyloid clearance, and also plays an important role in hippocampal neurogenesis. There are three common isoforms of ApoE in humans, including ApoE2, ApoE3, and ApoE4. The study indicated that overexpression of ApoE4 decreased adult neurogenesis in mice comparing with wild-type controls, while ApoE3 and ApoE2 expression promoted the proliferation and genesis of neural progenitor cells in the DG region (Koutseff et al., 2014). Moreover, ApoE4 can negatively affect adult hippocampal neurogenesis by modulating γ-aminobutyric acid interneurons and decreasing levels of neurosteroids (e.g., allopregnanolone). In contrast, ApoE3 can counteract chemokine-induced neuroinflammation and improve AHN (Suidan and Ramaswamy, 2019). Therefore, ApoE is expected to be a therapeutic target for AD (Chen et al., 2021).

In addition, by studying the “senile plaques” in the brain of AD patients, it was found that in addition to Aβ aggregation, there are many kinds of lipids in the senile plaques, such as ganglioside. Specific accumulation of monosialic acid gangliosides was found in the hippocampal subgranular region of a 12-month-old 5xFAD-AD mouse model, suggesting a potential relationship between the accumulation of plaque-associated gangliosides and damaged neural regeneration (Kaya et al., 2020).

### 3.2. Epigenetics

As technology improves, more and more studies show that epigenetic mechanisms, such as DNA methylation, histone post-translational modifications and microRNA-mediated post-transcriptional gene regulation, are involved in the pathogenesis of AD and thus influence the onset and progression of AD (Hernaiz et al., 2022; van Zundert and Montecino, 2022).

#### 3.2.1. DNA methylation

DNA methylation is an epigenetic mechanism that occurs by the covalent transfer of a methyl group to the C-5 position of the nuclear base cytosine to form 5-methylcytosine, which is involved in the regulation of gene expression (Moore et al., 2013). In order to investigate whether epigenetic alterations lead to late-onset AD, some researches focused on the DNA methylation status of the risk genes. The results showed that there is a decrease in CpG methylation at the APP gene promoter in neurons and glial cells, accompanied by an increase in APP gene expression, which in turn leads to an overproduction of Aβ (Tohgi et al., 1999). In addition, changes in DNA methylation also occur in other related genes, such as MCF2L, MAP2, ANK1, HOX3A, etc. (Gasparoni et al., 2018).

**FIGURE 2:**
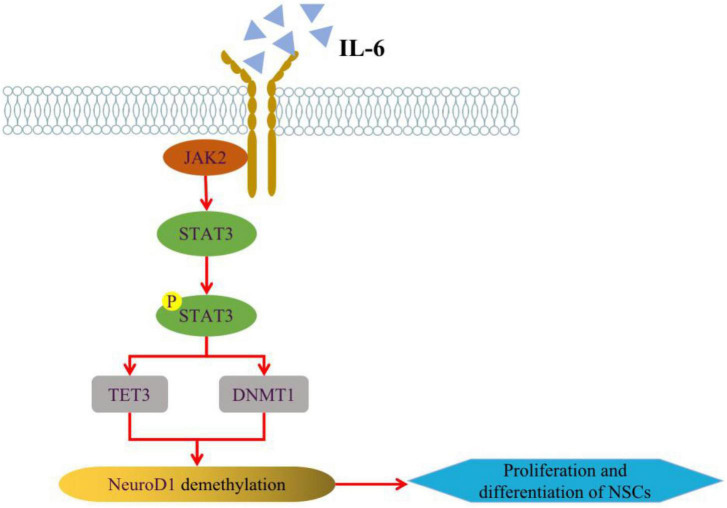
Regulation of IL-6/JAK2/STAT3 pathway on AHN. IL-6 can alter the expression of TET3 and DNMT1 in NSCs through JAK2/STAT3 signaling pathway, thus enhancing the expression of NeuroD1 (Neurogenic differentiation 1) gene through active or passive DNA demethylation, and regulating NSCs neurogenesis at the epigenetic level. Some of this illustration was started from a Scidraw Template.

Among the pathological features of AD, impairments in epigenetic mechanisms also lead to the generation of damaged neurons by neural stem cells, which will aggravate the loss of neurons and the defects of learning and memory (Li et al., 2016). DNA methylation of promoters can result in transcriptional repression through DNA methyltransferases (DNMTs) in neurodevelopment, while the deletion of DNMT1 in neural progenitors impairs the maturation and survival state of neurons, causing precocious astrocyte differentiation (Hutnick et al., 2009). And it was shown that the IL-6/JAK2/STAT3 pathway can regulate neurogenesis by improving DNMT1 in NSCs, promote DNA demethylation to regulate NSCs status at the epigenetic level, and further regulate AHN (Kong X. et al., 2019; Figure 2). The development of neural progenitor cells is an important node in the process of AHN, and mitochondria in neural progenitor cells play a crucial role in AHN (Gage, 2019). Whether in AD model mice or in Aβ-induced *in vitro* cell lines, the aggregation of Aβ can induce mitochondrial damage in neural progenitor cells and lead to the defect of AHN and cognitive function. In-depth mechanistic studies revealed that damage to mitochondria causes degradation of lysine demethylase 5A (KDM5A) in neural progenitor cells, which in turn result in AHN deficits. It may be because the genetic reduction of KDM5A expression reduces the transcription of BDNF in the brain, inhibits neural differentiation and leads to memory deficits (Bath et al., 2012; Kim et al., 2022). Additionally, the human neural progenitor cells induced from pluripotent stem cells were used to establish a simple *in vitro* adult neurogenesis model to study DNA methylation in the context of AD disease. And it found that the methylation levels of NXN genes were elevated and differential DNA methylation were observed in immature neurons by quantifying genes related to neurogenesis in the hippocampal region in the context of simulated AD (Blanco-Luquin et al., 2022).

#### 3.2.2. Histone modification

The appearance of neurofibrillary tangles and the aggregation of neural plaques in AD patients are some markers of abnormal histone post-translational modifications during the development of AD, and these abnormal modifications are closely related to the cognitive impairment of AD patients in the later stage. Histones are structural proteins that form DNA nucleosomes. Their N-terminal sequences protrude beyond the boundaries of nucleosomes and provide a surface for the recognition and subsequent interaction of proteins that regulate transcription. They usually participate in the expression and repression of target genes through chromatin modification. At present, histone modifications that have been studied extensively include acetylation, methylation and phosphorylation (Voigt and Reinberg, 2013; Audia and Campbell, 2016). Phosphorylation is one of the most common modifications in proteome biology, and approximately 30% of proteins undergo this modification by adding phosphate groups to serine, tyrosine and threonine residues. There is also growing evidence that phosphorylation modifications in many diseases alter the basic function of proteins and drive cell death signals (Humphrey et al., 2015). Tau protein, a microtubule-associated protein, has been proved to promote the formation of neurogenic fiber tangles by hyperphosphorylation in the brains of AD patients. The phosphorylated Tau protein accumulates excessively in GABAergic interneurons in the DG region of AD patients and mice, which impair the AHN by suppressing GABAergic transmission (Zheng et al., 2020). And this hyperphosphorylated Tau protein accumulation occurs mainly in the early and middle stages of AD. In the late stage of AD, the phosphorylated Tau protein gradually disappears from the nuclei of the granule cell of the DG region (Gil et al., 2022). Heme oxygenase was found to be neuroprotective in AD mice by modulating the levels of p–Ser9-GSKβ and p–Tyr216-GSKβ to improve the survival and proliferation capacity of NSCs and promote AHN in AD disease model (Si et al., 2020). In a mouse model of fragile X-chromosome syndrome, scientists found that neurogenesis could be improved by improving the level of histone acetylation modification to rescue cognitive deficits in mice (Li et al., 2018). In addition, H3K9me3, the ninth lysine on histone H3 is methylated and is a common inhibitory histone modification. Scientists have demonstrated that the expression and distribution of H3K9me3 changes dynamically in dentate gyrus of adult mouse and cultured adult hippocampal neurons, exhibiting high levels of expression early in neurogenesis. And silencing of H3K9 transferase mediated by retrovirus significantly impairs neural progenitor cell differentiation (Guerra et al., 2021). To understand the specific role of histone modifications in AD, more future studies are needed to be carried out.

#### 3.2.3. miRNAs

MiRNAs are a class of single stranded non-coding RNAs with about 19–23 nucleotides, which are important epigenetic and post-transcriptional regulators of mRNA complexity. Due to their small molecular weight, amphiphilic and high solubility, they have strong mobility and are ubiquitous in the central nervous system. They are the minimum information of nucleic acid signal molecules in eukaryotes described so far (Zhao et al., 2020). In recent years, a growing number of studies have shown that miRNAs play a key role in the development of many neurodegenerative diseases, and their role in the pathogenesis of AD involves the regulation of amyloid precursor protein (APP), BACE1, neuronal apoptosis and other factors (Deng et al., 2014). With the development of scientific technology, miRNA dysregulation has also been found to be associated with the development of AD (Rahman et al., 2020). The network analysis of brain miRNAs in AD patients at Braak stage III revealed that a large number of miRNAs were related with the immune system, cell cycle, gene expression, cellular stress response, nerve growth factor signal, Wnt signal and cellular senescence, such as miR-107, 26b, 30e, 34a, 485, 200c, 210, 146a, 34c, 125a. Among them, miR-200c, 146a, 34c, 125 expression is increased in the brains of AD patients, while miR-210, 485, 107 expression is decreased, and the remaining ones are dynamically changed with disease progression (Swarbrick et al., 2019).

As increasing studies have observed the dysregulation of microRNA expressions in AD, more researches have been conducted on their effect on AHN. MiR-9 is one of the most abundant microRNAs in mammalian central nervous system. It can promote neural differentiation of NSCs by targeting related genes to induce neurogenesis (Landgraf et al., 2007; Yoo et al., 2009). And it can inhibit Notch signal pathway (participating in the regulation of nervous system development and stem cell biological activity) in AD and promote NSCs to differentiate into neurons, thus promoting AHN (Li et al., 2017). The expression levels of miR-206 were increased in Tg2576 AD mice and AD patients. Inhibition of miR-206 expression in AD mice could improve Aβ deposition as well as BDNF expression levels, enhance hippocampal synaptic density and neurogenesis, and further improve memory function (Lee et al., 2012). MiR-351-5p is a pathogenic factor of hippocampal neural progenitor cells, which is widely expressed in AD patients. Inhibition of its downstream gene Miro 2 can induce a large number of mitochondrial divisions and lead to bad mitosis, which result in the loss of a large number of normal mitochondria and the death of hippocampal neural progenitor cells (Woo et al., 2020). MiR-132 is one of the consistently downregulated microRNAs in the development of AD. And it is an effective regulator of AHN. When miR-132 levels were restored in the hippocampus, the hippocampal AHN and associated memory deficits can be improved in AD mice (Walgrave et al., 2021). MiR-188-5p expression is also reduced in AD samples, while increasing its expression levels in primary hippocampal neurons and 5xFAD mouse neurons was found to further improve cognitive deficits by improving neuronal survival (Lee et al., 2016).

### 3.3. Neuroinflammation

In the brain of AD patients, neuroinflammation is one of the key pathological features. Microglia are the major inflammatory cells of the central nervous system, and under normal physiological conditions, their main functions are to support the integrity and survival of neuronal networks and to maintain brain homeostasis, development, etc. More and more studies have found that many AD-associated risk locus are unique or highly expressed in microglia, which suggests that microglia play an important role in the development of disease under the pathological conditions of AD (Pintado et al., 2017; Eshraghi et al., 2021; Lopes et al., 2022). The activation time of microglia is earlier than that of Aβ and Tau protein pathology in both brains of AD patients and AD mouse models (Tarkowski et al., 2003; Wright et al., 2013). And previous study also proved that Aβ can induce lysosomal damage and activate inflammatory corpuscles in microglia to mediate inflammatory reaction in central nervous system (Halle et al., 2008).

For AHN, only a small number of newborn cells in SGZ of DG are able to survive after phagocytosis by microglia and eventually integrate into local circuits, suggesting that microglia are critical for the homeostatic formation of the regenerative microenvironment within the brain for hippocampal neuroregeneration. More and more researches revealed that alterations in microglia status are critical for AHN. Interleukin-6 (IL-6), a pro-inflammatory cytokine known to regulate neurogenesis, has been reported to inhibit neurogenesis in neural stem cells *via* the JAK2/STAT3 signaling pathway (Kong X. et al., 2019). It was also demonstrated that photobiomodulation therapy can upregulate IFN-γand IL-10 expression levels in the brain of APP/PS1 and 3xTg AD model through JAK2-mediated signaling and STAT4/STAT5 signaling pathways, and induce improved microenvironmental conditions in the brain, which promotes AHN and reverse cognitive deficits (Wu et al., 2022). These findings are critical for potential therapeutic approaches in AD. In the early stage of AD, microglia can inhibit the progression of inflammation and assist in the clearance of Aβ to improve the intracerebral microenvironment, which is beneficial to AHN. However, with the development of AD disease, microglia will be affected by inflammatory factors that exacerbate the inflammatory reaction, and the interaction with astrocytes will further aggravate the neurotoxicity (Song et al., 2012; Gray et al., 2020). As a part of the innate immune system, the complement system plays an important role in the clearance of pathogens and cellular debris. In the healthy brain, complement affects neurodevelopment, neurogenesis, synapse formation and phagocyte recruitment, and protects the body from pathogens (Schartz and Tenner, 2020). However, in the process of AD disease, excessive activation of downstream complement will lead to the production of C5a, which can bind with the receptor and cause a series of negative reactions such as inflammation, injury and neuronal death (Ager et al., 2010). It has been shown that in AD, the C5a-C5aR1 signaling pathway can accelerate disease progression by enhancing the activation of microglia and exacerbating neuroinflammation (Carvalho et al., 2022). In the process of finding an effective treatment for AD, people found that sigma1 receptors (S1Rs) may regulate AHN, prevent Aβ-induced neurotoxicity and modulate the pathophysiology of AD, and have great potential as new targets for treatment (Penke et al., 2018; Ma et al., 2021). In an early AD model induced by Aβ 1–42, it was found that the use of two agonists of S1Rs–DMT and PRE084 not only significantly reduce the proliferation of highly reactive astrocytes, but also affect neurogenesis and the survival of mature neurons. Furthermore, as a presynaptic self-receptor, the histamine H3 receptor is highly expressed not only in neurons but also in microglia, and regulates the inflammatory response together with microglia (Naddafi and Mirshafiey, 2013). Inhibition of H3R decreases the activity of microglia, promotes a phenotypic shift from pro-inflammatory M1 to anti-inflammatory M2, and attenuates neuroinflammation. In addition, hippocampal neurogenesis was improved by enhancing histamine release, which in turn improved cognitive dysfunction (Wang et al., 2022).

## 4. Therapeutic potential of AHN in AD

As more and more researches demonstrate that AHN declines in AD patients, promoting nerve regeneration has emerged as a new target for AD treatment. It has been found that nerve regeneration can be promoted by reducing neuroinflammation. For example, the use of the anti-inflammatory drug minocycline can reduce neuroinflammation in the hippocampus by inhibiting microglia activation, which further promotes AHN (Lue et al., 2001). In addition, many studies support Chinese Herbal Medicine can be used for the routine treatment of AD (Cui et al., 2008; Mao et al., 2015; Chen et al., 2016; Yang et al., 2017). Stem cell transplantation can also reduce the effect of nerve inflammation on nerve regeneration and promote the emergence of new neurons, which is a promising method to change the pathology of diseases. However, due to some “side effects,” this type of treatment has not been widely used (Levenstein et al., 2006). Today, stem cell therapy using mesenchymal stem cells (MSCs) has emerged as a promising strategy for regulating the adult hippocampal niche. MSCs can migrate across the blood-brain barrier with the help of stimulants, generate and differentiate new neurons, and enhance memory function by secreting cytokines and trophic factors important for promoting and anti-inflammatory regulation (Noureddini and Bagheri-Mohammadi, 2021). Other studies have shown that enhancing neural progenitor cell differentiation can improve AHN. For example, fluoxetine can increase the proliferation of progenitor cells, while phosphodiesterase inhibitors can promote the differentiation of cells. Both of them can improve AHN to rescue cognitive dysfunction in mice with AD (Bartolome et al., 2018; Ou et al., 2018). Furthermore, lentivirus-mediated gene therapy for Wnt3 (upstream activator of the β-catenin pathway) overexpression can also promote AHN in the brains of AD patients (Lie et al., 2005). While NeuroD1 was shown to be an important target for repairing neuronal differentiation defects in the brains of AD patients, its overexpression promotes the transcription of genes related to neuronal differentiation, facilitating neuronal maturation and effectively improving spatial memory deficits in AD mice (Richetin et al., 2015; Pataskar et al., 2016). In recent years, studies at the molecular level have become increasingly advanced, and it has been found that miR-132 level changes are closely related to NSCs proliferation and differentiation besides Aβ pathology in AD mice. The overexpression of miR-132 can effectively regulate AHN, but whether it can be effectively applied to clinical needs more relevant researches (Walgrave et al., 2021).

**FIGURE 3:**
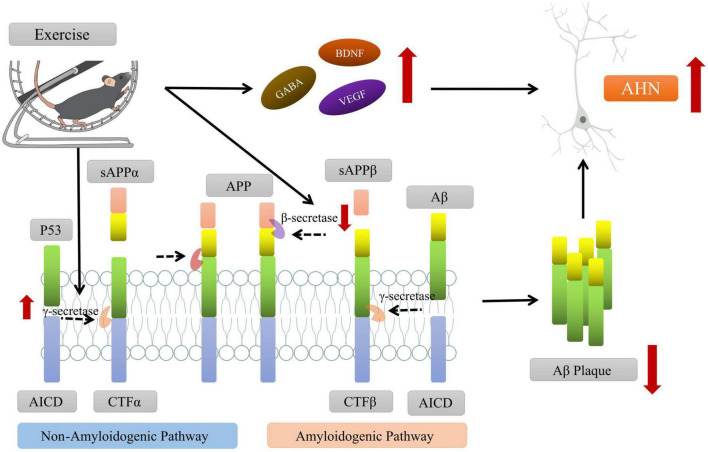
Exercise promoting the adult hippocampal neurogenesis (AHN) and improving the cognitive function of AD mice. On one hand, exercise intervention can promote the cleavage of amyloid precursor protein (APP) by affecting the levels of key secretory enzymes (such as β-secretase, γ-secretase, etc.) and reduce the aggregation of Aβ in brain. On the other hand, exercise can regulate the levels of neurotrophic factors (such as BDNF, VEGF, etc.), create a microenvironment conducive to AHN, and promote the survival and differentiation of new neurons. Some of this illustration was started from a Scidraw Template.

In addition to drugs, a growing body of research suggests that physical exercise may be an effective method for prevention or rehabilitation, which can improve the AHN and cognitive dysfunction in AD animal models through upregulating synaptic signaling pathways, downregulating inflammation-related genes and promoting neurogenesis (Maliszewska-Cyna et al., 2016; Santiago et al., 2022). To investigate the potential mechanisms between physical exercise and AHN, the scientists assessed the effects of treadmill exercise on the cognitive ability of AD mouse model. They found that exercise could modulate various neurotrophic factors (e.g., BDNF) and secretory enzymes (e.g., γ-secretase) that cleave APP through the non-β-amyloid pathway to reduce Aβ aggregation and improve the hippocampal microenvironment, which further promotes the survival, proliferation and differentiation of newborn neuron to enhance AHN and improve cognitive function of AD mouse (Vaynman et al., 2004; Inoue et al., 2015; Yu et al., 2021; Figure 3).

In a word, lots of studies have demonstrated the effectiveness of enhancing AHN to improve learning and memory cognitive performance in AD mice. However, more research is needed to improve the clinical use of this treatment.

## 5. Summary

In conclusion, the impairment of AHN during the development of AD is closely associated with learning memory and cognitive dysfunction in AD patients or animal models, which is widely confirmed. However, there are still some unknown factors that affect the consistency of the findings, which will require more research to verify its characteristic manifestations at different stages of AD development. In addition, as the pathogenesis of AD is increasingly elucidated and the mechanisms of AHN become clearer and clearer, the exact mechanisms of altered AHN in AD are gradually gaining more attention. Impaired energy metabolism, epigenetic mechanisms and neuroinflammation are important pathological features in the development of AD. And a growing number of studies have demonstrated that these pathological alterations have potential important link with the impairment of AHN. Overall, these findings provide strong evidence for our understanding of the interaction between the development of AD and AHN, which will help us to further identify targets that regulate neurogenesis as a potential approach to treat AD-related deficits. Although many studies have shown that promoting AHN can effectively improve cognitive deficits in AD, animal models, it is still challenging to put it into the clinical practice. Therefore, we need to improve our research to make it more applicable to AD patients, and provide new and effective therapeutic approaches.

## Author contributions

YZ, XW, and YL drafted the manuscript. YG assisted in data collection. RG, GZ, and QL helped to frame up and revised the manuscript. All authors have read and approved the final manuscript.
